# Rare complications related to lumen-apposing metal stent placement, successfully treated by endoscopic hand-suturing device.

**DOI:** 10.1055/a-2072-5740

**Published:** 2023-05-04

**Authors:** Yosuke Minoda, Nao Fujimori, Mitsuru Esaki, Shuzaburo Nagatomo, Yasuhiro Komori, Keijiro Ueda, Eikichi Ihara

**Affiliations:** 1 Kyushu University Faculty of Medicine Graduate School of Medical Science, Department of Medicine and Bioregulatory Science, Fukuoka, Fukuoka, Japan; 2 Kyushu University, Department of Endoscopic Diagnostics Therapeutics, Fukuoka, Fukuoka, Japan; 3 Kyushu University Faculty of Medicine Graduate School of Medical Science, Department of Gastroenterology and Metabolism, Japan

A 56-year-old man with a walled-off necrosis (WON) underwent endoscopic necrosectomy using a lumen-apposing metal stent (LAMS) (Hot Axios; Boston Scientific, Boston, Massachusetts, USA) ( Fig. 1 a ), which improved the abdominal inflammation within 1 month. However, 1 year after removal of the LAMS, the WON relapsed with abdominal pain and elevated C-reactive protein (CRP) levels. Gastroscopy revealed that the fistula caused by the LAMS placement persisted, with leakage of necrotic material. Fluoroscopy also revealed a colonic fistula associated with the WON in the sigmoid colon ( Fig. 1 b ). An ileostomy, in combination with drainage using a plastic stent into the WON and administration of antibiotics, failed to improve abdominal symptoms and normalize CRP levels; this was suggested to be attributed to the two fistulas. After percutaneous drainage of the WON, an over-the-scope (OTS) clip was applied to close these fistulas. However, the colon and gastric tissues around the fistula were too rigid to be pulled in the OTS clip. In this situation, we attempted to close the gastric fistula using a new endoscopic hand-suturing device (SutuArt; Olympus, Tokyo, Japan) and a suture needle with thread (V-lock; Medtronic, Dublin, Ireland) after cauterization of the gastric mucosa surrounding the fistula to promote wound healing ( Fig. 1 c–g , Video 1 ). One month later, the fistula was completely fixed with a normal covered mucosa ( Fig. 1 h ). This led to an improvement in abdominal pain and CRP levels.

**Fig. 1:**
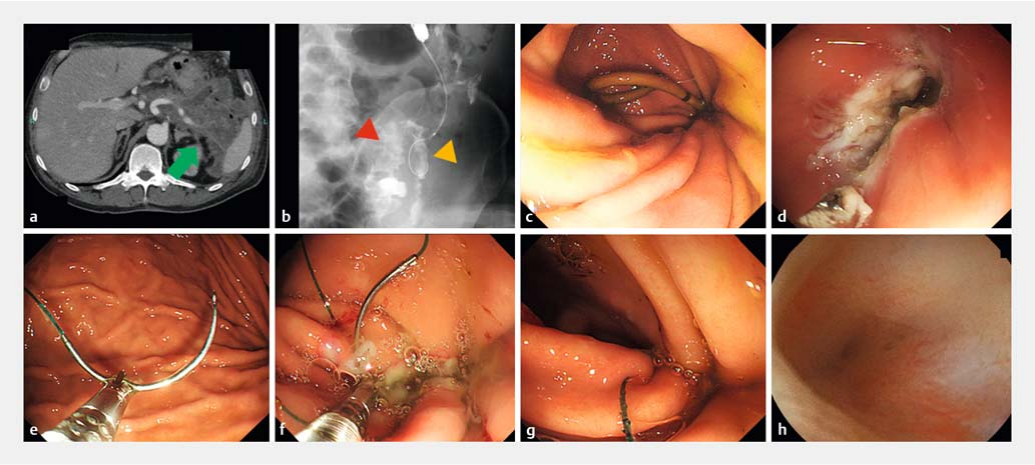
Steps to close the fistula associated with the placement of a lumen-apposing metal stent using an endoscopic hand-suturing device. **a** Computed tomography revealed the presence of the walled-off necrosis (WON) (green arrow). **b** Fluoroscopy revealed a colonic fistula associated with WON (yellow triangle) at the sigmoid colon (red triangle). **c** A plastic stent was placed to improve the relapsed WON and to close the fistula. **d** The gastric mucosa around the fistula was cauterized circumferentially using an electrical device. **e** A suture needle with thread was introduced into the stomach using an endoscopic hand-suturing device. **f** An endoscopic hand-suturing device and a suture needle with thread were applied to close the fistula. **g** The fistula was completely sutured with thread. **h** One month after endoscopic suturing, the fistula was completely fixed with a normal covered mucosa.

**Video 1** Steps to close the fistula associated with the placement of a lumen-apposing metal stent using an endoscopic hand-suturing device.


Usually, LAMS-associated fistulas are closed in their natural course 1 2 3 . We encountered a rare case in which a long-term remaining fistula resulted in WON relapse, which was successfully treated by closing the fistula using a new endoscopic suturing device. This hand-suturing device is a good option for closing LAMS-associated fistulas.

Endoscopy_UCTN_Code_CPL_1AH_2AG

## Notes

Endoscopy E-Videos

https://eref.thieme.de/e-videos
*E-Videos* is an open access online section of the journal *Endoscopy* , reporting on interesting cases and new techniques in gastroenterological endoscopy. All papers include a high-quality video and are published with a Creative Commons CC-BY license. Endoscopy E-Videos qualify for HINARI discounts and waivers and eligibility is automatically checked during the submission process. We grant 100% waivers to articles whose corresponding authors are based in Group A countries and 50% waivers to those who are based in Group B countries as classified by Research4Life (see: https://www.research4life.org/access/eligibility/ ). This section has its own submission website at https://mc.manuscriptcentral.com/e-videos
